# Polystyrene sulfonate is effective for enhancing biomass enzymatic saccharification under green liquor pretreatment in bioenergy poplar

**DOI:** 10.1186/s13068-022-02108-y

**Published:** 2022-01-21

**Authors:** Tian Liu, Peipei Wang, Jing Tian, Jiaqi Guo, Wenyuan Zhu, Yongcan Jin, Huining Xiao, Junlong Song

**Affiliations:** 1 International Innovation Center for Forest Chemicals and Materials and Jiangsu Co-Innovation Center for Efficient Processing and Utilization of Forest Resources, Nanjing Forestry University, Nanjing, 210037 China; 2 Department of Chemical Engineering, University of New Brunswick, Fredericton, NB E3B 5A3 Canada

**Keywords:** Lignocellulose, Lignosulfonate (LS), Sodium polystyrene sulfonate (PSS), Enzymatic hydrolysis, Quartz Crystal Microbalance (QCM), Multi-parametric Surface Plasmon Resonance (MP-SPR), Interaction mechanism

## Abstract

**Background:**

Water-soluble lignin (particularly lignosulfonate, LS) has been well documented for its significance on enzymatic saccharification of lignocellulose, though the promotion mechanism has not been fully understood. Much attention has been paid to natural lignin or its derivatives. The disadvantage of using natural lignin-based polymers as promoting agents lies in the difficulty in tailor-incorporating functional groups due to their complex 3D structures. To further improve our understanding on the promotion mechanism of water-soluble lignin in the bioconversion of lignocellulose and to pursue better alternatives with different skeleton structures other than natural lignin, herein we reported a synthetic soluble linear aromatic polymer, sodium polystyrene sulfonate (PSS), to mimic LS for enhancing the efficiency of enzymatic saccharification.

**Results:**

The role of PSS in enzymatic saccharification of pure cellulose and green liquor-pretreated poplar (GL-P) was explored by analyzing substrate enzymatic digestibility (SED) under different addition dosages and various pH media, along with LS for comparison. At the cellulase loading of 13.3 FPU/g-glucan, the glucose yield of GL-P increased from 53% for the control to 81.5% with PSS addition of 0.1 g/g-substrate. It outperformed LS with the addition of 0.2 g/g-substrate by 6.3%. In the pH range from 4.5 to 6, PSS showed a positive effect on lignocellulose saccharification with the optimum pH at 4.8, where the most pronounced SED of GL-P was achieved. The underlying mechanism was unveiled by measuring zeta potential and using Quartz Crystal Microbalance (QCM) and Multi-parametric Surface Plasmon Resonance (MP-SPR). The results confirmed that the complexes of cellulase and PSS were conjugated and the negatively supercharged complexes reduced non-productive binding effectively along with the improved saccharification efficiency. The thickness of PSS required to block the binding sites of cellulase film was less than half of that of LS, and the PSS adlayer on cellulase film is also more hydrated and with a much lower shear modulus than LS adlayer.

**Conclusions:**

PSS as LS analogue is effective for enhancing the biomass enzymatic saccharification of GL-pretreated poplar. PSS exhibited a severer inhibition on the enzymatic saccharification of pure cellulose, while a more positive effect on bioconversion of lignocellulose (GL-P) than LS. In addition, a much lower dosage is required by PSS. The dynamic enzymatic hydrolysis indicated PSS could prolong the processive activity of cellulase. The valid data stemmed from QCM and SPR expressed that PSS bound to cellulases and the as-formed complexes reduced the non-productive adsorption of cellulase onto substrate lignin more efficiently than LS due to its flexible skeleton and highly hydrated structure. Therefore, PSS is a promising alternative promoting agent for lignocellulose saccharification. From another perspective, the synthetic lignin mimics with controllable structures enable us to reach an in-depth understanding of the promotion mechanism of soluble lignins on enzymatic saccharification.

## Background

As an inexhaustible resource on earth, lignocellulosic biomass has been extensively explored for the production of clean energy and green chemicals [1–3]. To improve the enzymatic hydrolysis efficiency and save the high cost of enzymes, some enzymatic promoting additives were brought in the enzymatic saccharification system, including surfactants (Tween [4], polyethylene glycol (PEG) [5], cetyltrimethylammonium bromide (CTAB) [6], Silwet L-77 [7], etc.), non-catalytic proteins (e.g., bovine serum albumin (BSA) [8], peanut protein [9], and Amaranthus green proteins [10]), soluble lignin and derivatives [11–14].

It takes a long time to recognize the intricate functions played by lignin and derivatives in the process of enzymatic hydrolysis of lignocelluloses. Initially, lignin was always considered as an inhibition factor based on the facts of (1) pretreatment cannot remove all the lignin and the residual lignin on the surface of cellulose substrate occupies the adsorption sites belonging to cellulases via steric hindrance; (2) soluble lignin-derived compounds may restrain the activity of cellulases; and (3) last but not the least, the non-productive adsorption exists between lignin and cellulases [15–17]. In 2009, a novel SPORL process (the sulfite pretreatment to overcome recalcitrance of lignocellulose) was developed by Zhu’s group [18] to pretreat lignocellulose and they found that it facilitated the bioconversion of softwood with reduced energy consumption and low amounts of inhibitors generated. In this process, native lignin was sulfonated to form lignosulfonate (LS). Later on, they found that the hydrolysate (major component was LS) from SPORL pretreatment or even commercial LS had a positive effect on enhancing the enzymatic cellulose saccharification of SPORL-pretreated poplar and lodgepole pine by 25.9% and 31.8%, respectively, especially in the case that the substrate contained substantial residual lignin [12, 19]. Then Wang et al. [20] found that alkaline lignin marginally boosted enzymatic hydrolysis of silvergrass, poplar and masson pine less than 10%. LS is one of the by-products of sulfite pulping [21]. Nowadays, the sulfite pulp production keeps declining. Aro and Fatehi [22] introduced the sulfonic acid groups to the aromatic ring of kraft lignin and the product as prepared also had promoting effect. Recently Lou and co-workers grafted quaternary moieties [11, 23], phosphobetaine [24], and polyethylene glycol [25–27] onto lignin or LS skeleton. The resulting products promoted lignocellulose saccharification significantly and could act as effective carriers for cellulase recycling due to their pH responsiveness.

Hydrogen-bonding, hydrophobic interaction, and electrostatic attraction mainly contribute to the non-productive adsorption of cellulase and substrate lignin [15, 28]. Hydrophobic interaction can be tuned by adjusting the medium pH. Generally, pH 4.8–5.0 is considered as the optimum environment for cellulase work. However, Lan [29] and Lou [28] suggested that maximal substrate enzymatic digestion from hydrolysis of lignocellulose should be conducted at 5.2–6.0, even 6.2. However, in the LS promoting system, Zhu and co-workers showed that the bioconversion of substrate increased by 10% when the pH changed from 4.8 to 5.5[12]. This is attributed to the enhanced electrostatic repulsion and reduced non-productive binding of cellulases onto residual lignin since the elevated pH makes residual lignin less hydrophobic while cellulases carry more negative charges [28]. In addition, LS with different molecular weight (MW) and sulfonation degrees has different acceleration performances on bioconversion of lignocellulosic substrate. Lou et al. [30] reported that LS with higher MW had a stronger blocking effect on cellulase adsorption on residual lignin. Whereas Zhou et al.[13] reported that the LS sample with the lowest MW showed the best enhancement of 31% for the kraft lodgepole pine substrate while least significant for the SPORL-pretreated lodgepole pine; and this promoting effect disappeared in the case of LS with high MW and low degree of sulfonation [13, 31]. The controversy regarding the influence of LS MW on enzymatic promoting effect may be attributed to the variation of LS samples or LS fractions and substrates used. In another opinion, adding LS reduces non-productive adsorption through the formation of LS–lignin complexes and LS–cellulase complexes [32]. The sulfonate groups endow LS with negative charges and are inclined to adsorb on the amino groups of cellulase [19]. The strong interaction of LS with cellulases was confirmed by Wang et al.[33] by monitoring the formation of LS–lignin complexes in situ and real-time using Quartz Crystal Microbalance with Dissipation (QCM-D) and Surface Plasmon Resonance (SPR). Surprisingly, the promoting effect of LS was reversed with the pure cellulose as substrate [12, 19, 34]. Zheng et al.[32] attributed the adverse impact of LS on bioconversion of pure cellulose to the LS adsorbed on both cellulose and enzyme, in which the formed complexes impeded them from adsorbing. Overall, the promoting mechanism of soluble lignin and derivatives on the enzymatic hydrolysis of cellulose and lignocellulose is rather complicated and has yet to be fully understood.

**Fig. 1:**
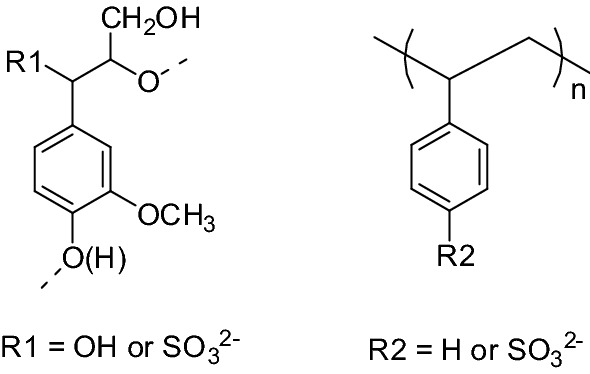
Molecular structure of lignosulfonate (LS, left) and polystyrene sulfonate (PSS, right)

Natural lignin’s derivatives, e.g., LS, alkaline lignin, and sulfonated alkaline lignin, are all rich in aromatic compounds with 3D networks. Sodium polystyrene sulfonate (PSS) is a linear aromatic polymer and prepared from a free-radical polymerization of sodium p-styrenesulfonate or sulfonating polystyrene [35]. PSS has been used as flocculants, anti-statics, emulsifiers, catalysts for a variety of applications associated with ion-exchange resin, stabilizing agents, Janus nanoparticle and biomaterial, etc. [36]. Both of their monomer structures are shown in Fig. 1. Compared with lignin and derivatives, PSS can be tailor-synthesized with controllable MW, charge density, and other properties. The hypothesis we proposed in this situation is that aromatic compounds, even linear PSS can promote the enzymatic hydrolysis via the formation of aromatic compound–enzyme complexes by π–π stacking interaction, and the resultant complexes weaken the non-producing adsorption of residual lignin on the substrate. As a mild alkali pretreatment method to remove some lignin and a small portion of hemicelluloses, GL pretreatment mainly used two chemical reagents, Na_2_S and Na_2_CO_3_ which are recycled from the kraft pulping process, to enhance the enzymatic hydrolysis productivity of hardwood [37, 38]. The benefits of this process are it reduces the amount of poisonous or erosive substances, such as furfural, acetic acid, and metal ions which may inhibit the subsequent fermentation or damage the pretreating equipment [39]. The enzymatic saccharification enhanced by LS is more pronounced for substrates subjected to kraft or alkaline pretreatment if compared with other pretreatments [13]. In this investigation, PSS was added into the enzymatic hydrolysis system to evaluate its performance, along with LS for easy comparison. For the first time, we found that PSS can mimic LS to enhance enzymatic hydrolysis efficiency for lignocellulose, and perform much better even at a lower dosage. Moreover, to better understand the interactions between PSS and cellulases from a fundamental point of review, QCM-D and MP-SPR were utilized to reveal the adsorption behaviors in an attempt to elucidate the mechanism.

## Results

### Structure characterizations of PSS

**Fig. 2:**
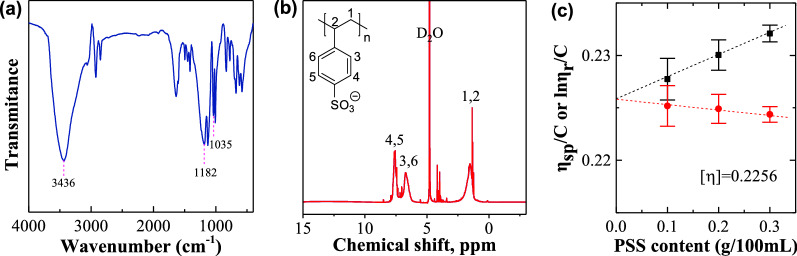
Chemical characterizations of PSS by **a** FT-IR spectra, **b** ^1^H-NMR spectra, **c** viscosity molecular weight. The black symbols were obtained from Huggins formula and the red symbols was obtained from Kraemer formula in **c**

The commercial PSS sample was characterized by FT-IR, ^1^H-NMR, and viscosity molecular weight (*M*_ƞ_), and the results are presented in Fig. 2a–c. In Fig. 2a, the O–H stretching vibration is observed at band centered at 3436 cm^−1^, and the sulfonic acid group and the S=O characteristic absorption peaks in sulfonate are observed at 1182 cm^−1^ and 1035 cm^−1^ [40]. From the 1H-NMR spectra shown as Fig. 2b, the proton peak assignment from different carbon is presented in the chart. The resonance from 6.4 to 7.9 ppm is due to the para-substitution on the benzene ring [41]. The MW of PSS was assessed by intrinsic viscosity [42]. The intrinsic viscosity of PSS was determined as shown in Fig. 2c, and the value was determined to be 0.2256 g/100 mL. *M*_ƞ_ of PSS was calculated based on Eq. (1) to be 61,080, which agreed with the value (~ 70,000) claimed by the supplier very well since viscosity-averaged molecular weight is usually less than the weight-averaged molecular weight. In sum, the structure of PSS used in this investigation was well-defined.

### Chemical composition of poplar variants with green liquor pretreatment and the effect of PSS dosage on the enzymatic saccharification

As shown in Table 1, the solid recovery was 84%, while most glucan (97%) in the poplar was reserved in the substrate. After GL pretreatment, the poplar lignin had high S/G ratios because of breaking amounts of β-O-4 linkages and G unit and had low esterified p-hydroxybenzoic acid, which improved enzymatic saccharification efficiency [43]. GL pretreatment reduced the crystalline index and polymerization degree of cellulose as well, ultimately increased substrate porosity to provide cellulase with the more contact area. The enhanced cellulase accessibility benefited the bioconversion rate [39, 44].

**Table 1:** The main components of poplar and GL-P (based on raw material)

Materials	Solid recovery (%)	Carbohydrate (%)			Lignin (%)		Ash (%)
		Glucan	Xylan	Ara + Man^b^	KL^c^	ASL^d^	
Poplar	–	43.0 ± 0.2	15.0 ± 0.2	2.1 ± 0.1	22.0 ± 0.9	2.4 ± 0.5	0.69 ± 0.01
GL-P^a^	84.00	39.4 ± 1.2	11.6 ± 0.1	1.6 ± 0.1	20.5 ± 0.3	2.5 ± 0.04	1.97 ± 0.04

^a^Green liquor-pretreated poplar, ^b^Arabinan + mannan, ^c^Klason lignin, ^d^Acid-soluble lignin

**Fig. 3:**
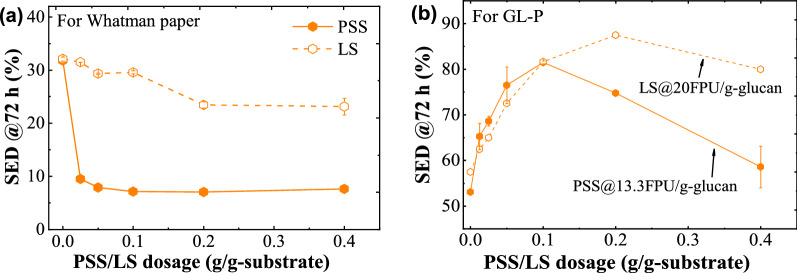
Enzymatic digestion rate of **a** pure cellulose and **b** green liquor-pretreated poplar (GL-P) after adding different dosages of PSS or LS. The enzymatic hydrolysis was conducted in acetate buffer (pH 4.8) and continued for 72 h. The cellulase dosage in a was 6.3 FPU/g-glucan while 13.3 FPU/g-glucan in b. The data of open symbols in b were adopted from Ref [45], in which enzyme loading was 20 FPU/g-glucan

To verify whether the PSS has a similar function as LS or not, different loadings of PSS were added in the enzymatic hydrolysis system to evaluate its performance. In terms of its performance on pure cellulose enzymatic hydrolysis (i.e., Whatman filter paper), the substrate enzymatic digestibility (SED) or glucose yield was 32.0% without PSS or LS addition (Fig. 3a) at the enzyme loading of 6.3 FPU/g-glucan. When 0.05 g/g-substrate LS was put in, the glucose yield dropped to 29.3%. The enzymatic digestibility was decreased with the LS increment. It reduced to 23.5% and 23.2% for the LS dosages of 0.2 and 0.4 g/g-substrate, respectively. From the work by Wang et al. [12], their enzymatic hydrolysis efficiency after 72 h decreased from 74.0% to 66.6% with the addition of 0.25 g/g-substrate commercial LS at an enzyme loading of 15 FPU/g-glucan. Therefore, the inhibition effect of LS on pure cellulose saccharification was consistent quite well with theirs in trend. Yet the effect of LS on enzymatic saccharification progress was closely bound up with MW and sulfonate group. As investigated by Lou et al. [31], LS with low MW and high sulfonate groups tends to promote the enzymatic hydrolysis of pure cellulose, whereas LS with high MW and low sulfonate groups induces the inhibition to some extent. However, PSS lowered the glucose yield dramatically to 9.5% even at the dosage as low as 0.025 g/g-substrate. With the PSS addition increased to 0.2 g/g-substrate, the glucose yield was gradually reduced to 7%. From this point of view, PSS exhibited a much severer inhibition on the enzymatic hydrolysis of pure cellulose than LS did. This may be attributed to its much higher MW (*M*_ƞ_ = 61,080), compared with that of LS (~ 10,000).

**Fig. 4:**
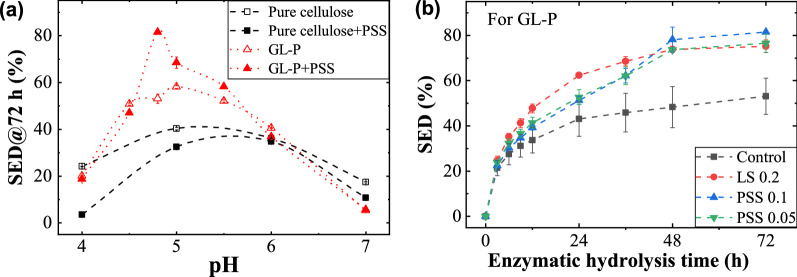
The effect of pH enzymatic hydrolysis process (**a**) and the dynamic enzymatic hydrolysis for 72 h (**b**). Enzyme loading was 6.3 FPU/g-glucan for Whatman paper while 13.3 FPU/g-glucan for GL-P saccharification. PSS dosage in a was 0.1 g/g-substrate. The buffer used in a was disodium hydrogen phosphate–citric acid buffer, while acetate buffer in **b**

The glucose yield of enzymatic hydrolysis of GL-P is depicted in Fig. 3b. As can be seen, the yield was only 53.0% in the absence of any PSS or LS, but increased significantly to 76.5% even at a very low dosage of PSS (i.e., 0.0125 g/g-substrate) under the enzyme loading of 13.3 FPU/g-glucan. Increasing PSS dosage led to the higher glucose yield and reached the highest (81.5%) at the PSS dosage of 0.1 g/g-substrate. However, when more PSS was added, the glucose yield started to decline, as observed in the cases of PSS dosages at 0.2 and 0.4 g/g-substrate. The promotion effect induced by PSS appeared to be similar to LS reported by Wang et al. [45], and their data are plotted in Fig. 3b for comparison. In their case, the highest glucose yield of GL-P reached 88.0% under the enzyme loading of 20 FPU/g-glucan with LS addition of 0.2 g/g-substrate. Even though their highest glucose yield was better than the value we obtained, it has to be noted their enzyme loading was 50% more than the enzyme loading in the current PSS system. When the dosage was less than 0.1 g/g-substrate, it can be observed the enzymatic hydrolysis rate induced by PSS was a little higher than that by LS at the same dosage, despite the enzyme loading difference. Under the same enzyme loading of 13.3 FPU/g-glucan, LS at its optimal dosage of 0.2 g/g-substrate led to the glucose yield of GL-P at 75.2% (Fig. 4b), which was inferior to the yield resulting from PSS at half the dosage. What’s more, the best glucose yield of GL-P obtained by PSS addition was much higher than that of poplar after SPORL pretreatment with SPORL hydrolysate addition (52.5%) [46]. In terms of overall performance and the reduced dosage, PSS exhibited better-promoting performance on the lignocellulose saccharification than LS.

### The effect of pH and the dynamic enzymatic hydrolysis

The system pH is a key factor influencing enzymatic saccharification of all kinds of substrate, especially with the participation of promoting additives [12, 28]. As depicted in Fig. 4a, enzymatic hydrolysis of Whatman paper and GL-P with and without the addition of 0.1 g/g-substrate PSS were conducted in the pH range from 4.0 to 7.0. Firstly, for pure cellulose, PSS inhibited the SED of Whatman paper in the whole range of pH from 4.0 to 7.0, particularly pronounced in the low pH range. The work reported elsewhere on LS [31] also showed that the optimal pH was changed with the MW and sulfonate content of LS. Meanwhile, the enzymatic saccharification of pure cellulose was enhanced especially by the LS of low MW and high content of sulfonate (or hydrophilic) groups which facilitate the binding with cellulase. Compared with LS, the sulfur content of PSS used in this work is relatively high (9.68 wt%), while the MW is also higher, compared with those of LS. As a result, the better performance of PSS in cellulose enzymatic hydrolysis can be anticipated.

In terms of the promoting performance for GL-P enzymatic hydrolysis, it was found on the contrary PSS promoted the SED of GL-P in the pH range between 4.5 to 6, particularly pronounced at the pH close to 4.8. The SED was improved from 53.1%, 62.1% and 52.1% to 81.5%, 66.5% and 58.2% when pH was 4.8, 5.0 and 5.5, respectively. Lou et al. [28] and Wang et al. [12] reported that LS exhibited a promotion effect at elevated pH, which was ascribed to the dissociation of phenolic groups of lignin (LS and residual lignin) and strong electrostatic repulsion between LS–cellulase complexes and residual lignin. In our case, there are no phenolic groups in PSS and the abundant sulfonate groups remain negative-charged in the pH range tested. Therefore, the pH effect on PSS-promoting behavior is not significant as that for LS. The optimal pH range for cellulases also remained unchanged.

To compare the effect of PSS and LS on enzymatic hydrolysis of lignocellulose, the glucose yield of GL-P after PSS/LS addition was plotted again enzymatic hydrolysis time and the results are shown in Fig. 4b. Since PSS processed a severer inhibition to the pure cellulose enzymatic hydrolysis, a lower PSS dosage at 0.05 g/g-substrate was used as well. The glucose yield of GL-P was increased obviously from 53.1% to 75.2% and 81.5%, respectively, with LS and PSS applied. Even for the low PSS dosage (0.05 g/g-substrate), its glucose yield after 72 h also reached 76.5%, which was slightly higher than that of LS at a fourfold dosage. After fully enzymatic hydrolysis for 72 h, the enzymatic digestibility of GL-pretreated substrate in the presence of additives followed the order of PSS 0.1 > PSS 0.05 > LS 0.2 > Control. Lou et al. [30] reported a much higher glucose yield when LS was applied as an additive. Compared with the results in our case, the substrate they used was the mixture of Whatman filter paper and enzymatic hydrolysis lignin. Even though their substrates contained similar chemical compositions as ours, the lignin chemistry and its distribution in the fiber should be quite different from the lignocellulose used in this work. The enzymatic hydrolysis lignin they used from steam explosion-pretreated corn stover, which was different from GL-pretreated poplar lignin had low S unit lignin apart from breaking β-O-4’, aryl-ether linkages [47].

Interestingly, when the enzymatic digestibility was examined at the period less than 48 h, a different order was observed. For example, for the enzymatic digestibility of 24 h, the order followed LS 0.2 > PSS 0.05 > PSS 0.1 > Control, which was opposite with the order of 72 h. The results indicated both additives of LS and PSS could promote enzymatic efficiency when compared with the control. However, if comparing enzymatic digestibility between LS and PSS additives, it can be found that the enzyme cocktails of cellulases and PSS showed a lower enzyme activity in a short processing period (less than 48 h), but with a prolonged processive time (beyond 48 h). This unique and interesting phenomenon was noticed for the first time in the current, which has not been reported yet in previous work. It remains unclear why PSS lowers the enzyme activity but with a longer processive time than LS, which is worthy of further investigation. Although PSS has resulted in an increased enzymatic digestibility, there is still a bottle neck of prolonged process time (enzymatic hydrolysis duration) that remains unaddressed.

### Interactions between PSS and cellulase

To investigate the effect of PSS on cellulase, the zeta potentials of the cellulases and the mixtures of cellulase with various loadings of PSS were measured and the results are given in Table 2. The cellulase used in this investigation had a slight zeta potential of 1.89 ± 0.05 mV. When PSS was added into the system and bound to cellulase molecules, all the mixtures of cellulase with various loadings of PSS exhibited negatively charged due to the strongly charged sulfonate groups of PSS, which rendered the complexes of cellulase and PSS with negative charges. With the increment of PSS addition from 0.05 to 0.10 g/g-substrate, the zeta potential of the mixtures dropped from − 28.95 to − 31.37 mV. Very interestingly, with further PSS increment to 0.15 and 0.20 g/g-substrate, the zeta potential of cellulase–PSS complex started to recover some extent to − 25.55 and − 21.53 mV, respectively. The most negatively charged mixture was achieved for the PSS dosage of 0.10 g/g-substrate. This agreed with the previous SED results; and the negatively supercharged cellulases rendered them lignin-resistant, as addressed by Whitehead et al. [48].

**Table 2:** Zeta potential (ζ) of cellulase and the mixtures of cellulase and PSS

Samples	Cellulase	Cellulase + 0.05 g/g-substrate PSS	Cellulase + 0.10 g/g-substrate PSS	Cellulase + 0.15 g/g-substrate PSS	Cellulase + 0.20 g/g-substrate PSS
ζ (mV)	1.89 ± 0.05	− 28.95 ± 1.88	− 31.37 ± 0.17	− 25.55 ± 0.55	− 21.53 ± 1.37

**Fig. 5:**
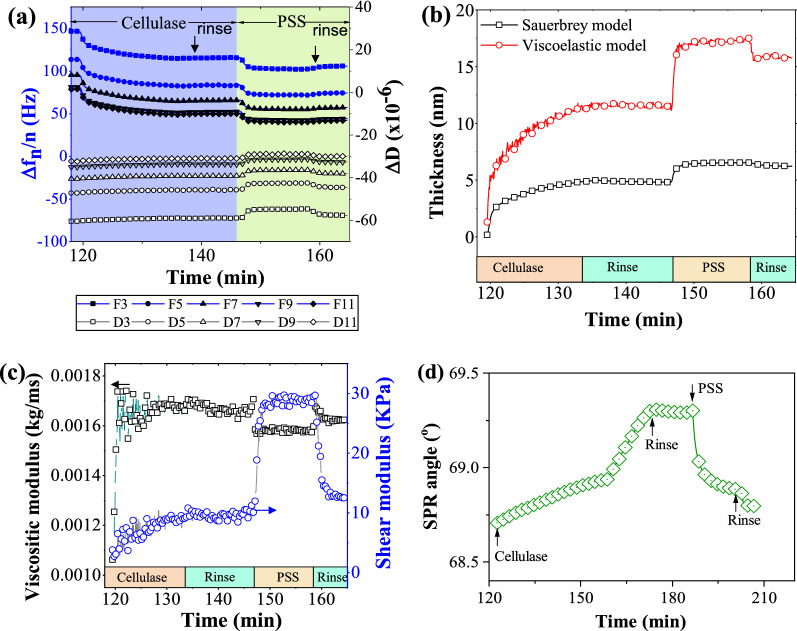
The formation of complexes of cellulase–PSS monitored by QCM-D E4 and MP-SPR in situ and in real-time and the fitting results. **a** The overtone data of frequency and energy dissipation changes during cellulase immobilization and complexes of cellulase–PSS formation monitored by QCM-D in situ and real-time; **b** thickness and **c** viscositic and shear moduli of cellulase and PSS films gradually deposited on the surface of the gold sensor, obtained by analyzing the QCM-D data; **d** SPR angle changes during cellulase immobilization and complexes formation of cellulase–PSS monitored by MP-SPR. The medium was acetate buffer (pH = 4.8, 0.05 M). The QCM and SPR chambers were thermostated at 25 ± 0.01 °C during the whole experiment

The interaction between cellulase and PSS monitored by a QCM-D E4 is presented in Fig. 5a. Initially, the cellulase was immobilized on the gold surface of QCM sensors and then the interaction between PSS and cellulase was monitored in situ and in real-time (the data not shown in the chart). The cellulase solution was injected into the chamber at 119 min when the frequency (Δf) and the dissipation (ΔD) became smooth or steady. Once the PSS solution flowed into the chamber at 145.3 min, the Δf_3_ was dropped from + 116.3 Hz to + 100 Hz, and it recovered 5 Hz to + 105 Hz after buffer rinse. In the meanwhile, the ΔD_3_ increased mildly by about 1.25 × 10^−6^. This adsorption process proceeded relatively fast, taking approximately 17.5 min. Regarding the interaction between cellulase and LS, the Δf_3_ dropped by ca. 7.8 Hz and the ΔD_3_ raise ca. 1.43 × 10^−6^ in the same medium [33]. It can be seen that PSS has a similar interaction as LS does to form complexes with cellulase when they contact in solution.

Based on the QCM-D overtone data, the thickness, viscositic, and shear moduli of cellulase and PSS films were fitted with Sauerbrey and viscoelastic model [49, 50], and the results are depicted in Figs. 5b and 5c. Since Sauerbery always underestimates the film thickness when the film is viscoelastic and cellulase layer and the formed complex layer were indeed viscoelastic, thereafter the thickness was referred to as viscoelastic thickness. The thickness of cellulase film was 11.56 nm, which was quite close to the value (11.7 nm) reported previously by Wang et al. [33]. This demonstrated the cellulase immobilization on QCM sensors was repeatable and the protocol was reliable. When the stable complexes of cellulase and PSS were formed and subjected to buffer rinsing, the thickness of the PSS layer was 4.22 nm, which was a little bit thicker than that of LS film (i.e., 3.4 nm) adsorbed on the cellulase film (Table 3) [33]. Figure 4c demonstrates the viscositic and shear moduli real-time change with the formation of the cellulase and PSS films. The viscositic and shear moduli of cellulase adlayer were 0.00165 kg/ms and 9.7 kPa, respectively. When the PSS solution was loaded, the viscositic moduli dropped slightly to 0.00157 kg/ms and the shear moduli increased significantly to 28.7 kPa. The viscositic moduli of PSS adlayer recovered to 0.00162 kg/ms and the shear moduli dropped to 12.66 kPa after buffer rinse. Compared with the values obtained for cellulase and LS, the viscositic moduli of both were quite close, whereas the shear moduli of the complex of cellulase and PSS were much lower than that of the complex of cellulase and LS (Fig. 5c). This may be attributed to the difference in chemical structure between PSS and LS, since PSS exists as linear and random coils, whereas LS presents as rigid spheres in solution.

**Table 3:** Thickness and coupled water of PSS/LS adlayer on cellulase film

Layer	QCM* (nm)	SPR** (nm)	Coupled water (%)
PSS	4.22	0.2	95.3
LS***	3.4	0.73	78.5

*The data presented in Fig. 4a fitted by the software Q-Dfind provided by Biolin Scientific Corp.; **The data presented in Fig. 4d simulated by the software WinSpall version 3.02; ***The data of LS were cited from Ref. [50]

Based on the principles of QCM-D, it responds to the weight change on the surface of the sensor. As a consequence, the water molecules moved with the adsorbed layer are also considered. To further study the interaction between cellulase and PSS, surface plasmon resonance (SPR) based on optical technology was employed and it only measures the “dry” mass on the sensor. SPR angle changes with cellulase immobilization and PSS loading are depicted in Fig. 5d. The SPR angle increased with the cellulase binding on the gold sensor. When the PSS solution was injected in, the SPR angle decreased obviously, which is attributed to the low refractive index of PSS (n = 1.38). The fitted thickness of PSS with dry mass adsorbed on enzymes acquired by SPR was 0.2 nm (Table 3). This is much less than the value of LS adsorbed on cellulase film (0.73 nm). By comparing the thickness obtained by QCM-D and SPR techniques, the coupled water can be derived and the values are reported in Table 3. The results showed that the coupled water of PSS is as high as 95.3%, indicating it is super highly hydrated, while this value is only 78.5% for LS adlayer in the same condition. This can be attributed to the difference in sulfonating degree for PSS and LS samples. And the difference in hydration between PSS and LS layers also can explain why PSS layer possessed a much lower shear modulus than LS adlayer did.

### Mechanism of PSS promoting enzymatic saccharification

**Fig. 6:**
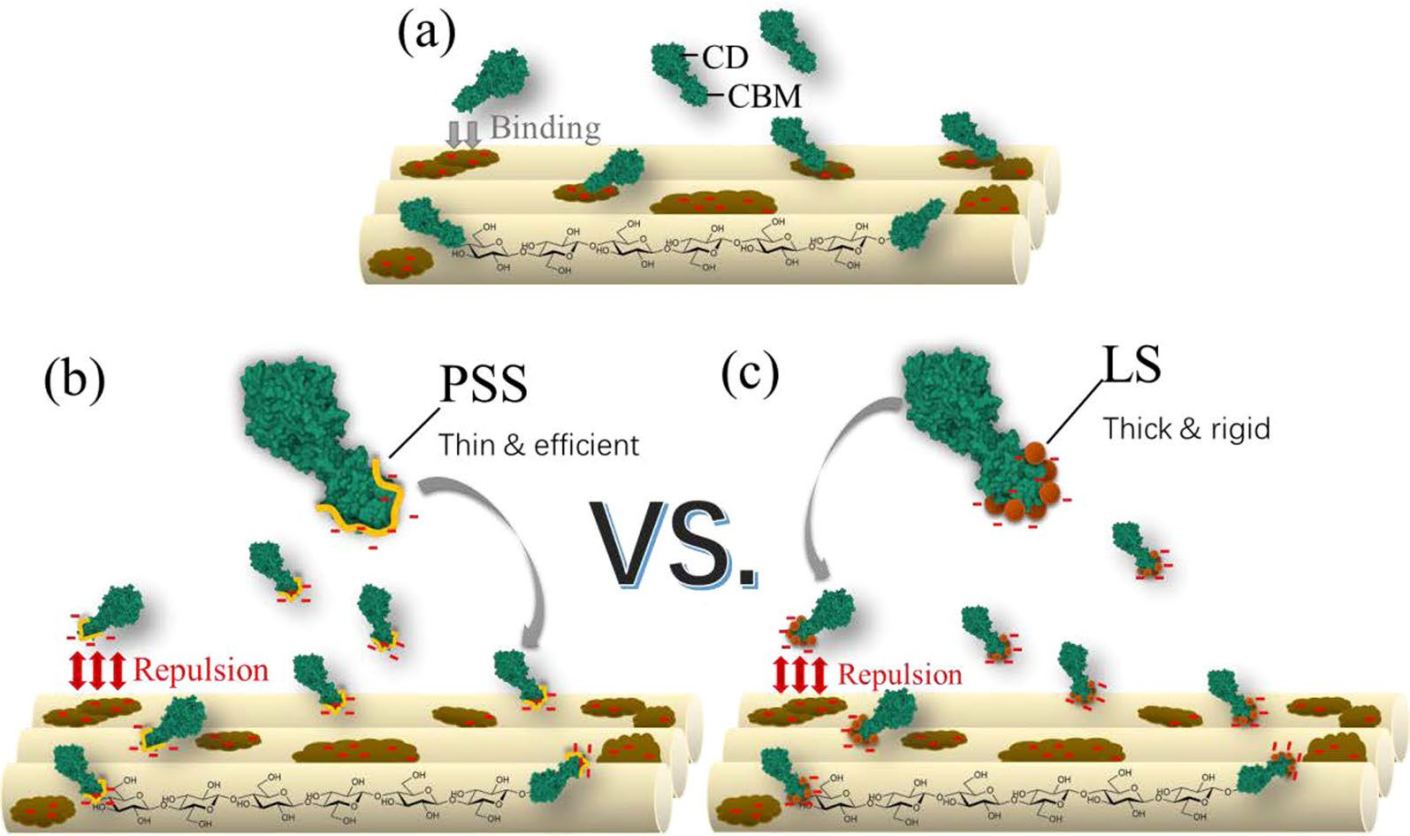
Proposed promoting mechanism of PSS on enzymatic saccharification of GL-P: **a** non-productive binding of cellulases on residue lignin in normal enzymatic saccharification system; **b** PSS’s promoting enzymatic saccharification of GL-P by reduced non-productive binding of cellulases on residue lignin (**c**) by the comparison with the addition of LS to highlight the importance of polymer’s morphology on the interactions between cellulase molecules and promoting additives

The carbohydrate-binding module (CBM) plays an important role in cellulose enzymatic hydrolysis [51] and also in lignin-binding since lignin and derivatives prefer to bind to the CBM portion of cellulases [52, 53]. To wrap it up, the PSS promoting mechanism for lignocellulose enzymatic saccharification is illustrated in Fig. 6. In a normal enzymatic hydrolysis scenario without promoting agent presence (Fig. 6a), some cellulase molecules perform normally to bind to cellulose surface by their CBM, while others are inevitably and irreversibly bind to the residual lignin due to the strong interactions between lignin and CBM, and the called non-productive binding occurs [52, 53]. When PSS is introduced into the system, as depicted in Fig. 6b, PSS molecules preferentially bind to the CBM of cellulases, and the formed complexes carrying more negative charges inhibit their non-productive binding to residual lignin due to the electrostatic repulsion, since residual lignin after GL pretreatment carries negative charges. As such, the amounts of cellulases that can bind to the cellulose surface increase proportionally. If there is no residual lignin present as in the case of Whatman filter paper, no such competitive adsorption occurs and therefore, only inhibition will be observed. Both scenarios are shared by LS and PSS.

Regarding the difference between LS and PSS, many studies confirmed that LS molecules present spheroidicity or dish structure in solution due to its 3D networks [54–56]; while PSS molecules take coiled conformation under moderately salted solution [57–59]. PSS exhibits a much-extended conformation in aqueous solution than that of LS (Fig. 6c). Therefore, the adlayer of PSS exhibited much lower shear moduli than those of LS (Fig. 4c). In addition, the high sulfonating degree of PSS renders it highly hydrated (Table 3). Due to its soft and flexible conformation, PSS can provide more binding sites to cellulases than LS in a given mass, representing the much lower adsorbed thickness monitored by SPR (Fig. 4d and Table 3). That is the underlying reason why PSS promotes lignocellulose enzymatic saccharification more efficiently than LS, especially at a lower dosage. However, the prolonging processive action of cellulases with the addition of PSS remains unresolved, and this makes the PSS-promoting mechanism for lignocellulose saccharification more profound and further investigation is desired.

## Conclusions

Synthetic linear PSS could imitate or mimic soluble lignin LS to enhance the efficiency of lignocellulose enzymatic saccharification and even perform more efficiently with a lower dosage. The interaction revealed by QCM-D and MP-SPR demonstrated that PSS had only less than half adsorbed thickness on cellulase film and the adsorbed PSS layer possessed much lower shear moduli than that of LS. This explains why PSS was more efficient to promote the lignocellulose saccharification at a lower dosage due to the flexible PSS chains and its highly hydrated nature. This study highlighted the importance of polymer/lignin structure to the synergic performance during lignocellulose saccharification. Since MW, charge density, and spatial structure of synthetic PSS can be readily controlled via copolymerization, the approach developed in this work offers us more opportunities to unveil the relationship between the structure of the soluble aromatic polymer and its promoting performance for lignocellulose saccharification and to identify the governing factors associated with the complexes formed between cellulase and promoting additives from a fundamental perspective.

## Experimental

### Materials

Poplar wood (triploid *Populus tomentosa*) was collected from Jiangsu, China, and cut into wood chips. Reax® 85A (LS, *M*_w_ ~ 10,000, sulfidity ~ 3.28%), from sulfonating kraft lignin was purchased from Nanjing Shanhu Chemical Co. Ltd (Nanjing, China). Sodium polystyrene sulfonate (PSS, *M*_w_ ~ 70,000, sulfidity ~ 9.68%) was purchased from Aladdin Corp. (Shanghai, China). Hydrolytic enzyme Cellic® CTec2 was provided by Novozymes (Franklinton, NC, USA). The QCM-D sensors with gold surface (Product number QSX 301) were supplied by Biolin Scientific Co. Ltd (Gothenburg, Sweden). SPR sensors were supplied by BioNavis Co. Ltd. (Tampere, Finland). All other chemicals, e.g., Na_2_CO_3_ and Na_2_S (AR, ≥ 98%) were purchased from Nanjing Chemical Reagent Co., Ltd. (Nanjing, China) and used without further purification.

### Characterizations of PSS

#### Viscosity molecular weight (*M*_ƞ_) of PSS

Dilute solutions with different contents of PSS in the buffer of 0.2 M acetic acid and 0.1 M sodium acetate were prepared, making sure that the polymer was extended fully in the buffer. The viscosity of the dilute PSS solution was determined by using an Ubbelohde viscometer (0.7 ~ 0.8 mm, Nanjing, China) in the water bath at 25 °C. The flow time of the buffer and the PSS solutions in the viscometer was recorded. Each solution was tested no less than three times and the error value no more than 0.2 s. The viscosity molecular weight of PSS (M_ƞ_) was calculated as Eq. (1) [60]:

1 $$\left[ \eta \right] = 0.001908 - 1 \times {\text{M}}_{\eta } ^{{0.851}} ,$$

where [ƞ] represents intrinsic viscosity.

#### Fourier transform infrared (FT-IR) and nuclear magnetic resonance (NMR) spectra of PSS

For FT-IR analysis, PSS was acquired on Bruker VERTEX80 spectrometer (Karlsruhe, Germany). Before testing, the PSS mixed with KBr was ground until the particle size was smaller than 2 μm and tested from 400 to 4000 cm^−1^. The scans were 32 times and the resolution was 4 cm^−1^.

The ^1^H-NMR spectra of PSS were recorded on Bruker 600 MHz spectrometer (Karlsruhe, Germany) with the pulse of zg30. Approximately 50 mg sample dissolved in 1 mL D_2_O for the test preparation.

### Poplar wood chips pretreated with green liquor

Poplar wood chips were pretreated under green liquor (GL) to prepare the pretreated substrate for enzymatic hydrolysis as described by Jin et al. [37] Parameters adopted in this pretreatment were: total titratable alkali (TTA) charge (in terms of Na_2_O) based on oven-dry (o.d.) of 20%, sulfidity of 25%, and solid-to-liquor ratio (g/mL) of 1:4. The poplar wood chips were impregnated for 30 min at 80 °C. Then the temperature was raised to 160 °C with the rate of 2 °C/min and kept at the maximum temperature for 1 h. When the pretreatment finished, the pulp was washed with tap water to remove residual chemicals and was defibered using a PFI refiner. Carbohydrate and lignin contents before and after GL pretreatment were analyzed by the NREL protocol [61], and the results are given in Table 1.

### Enzymatic hydrolysis and glucose analysis

Whatman filter paper was used as the representative of pure cellulose. The conditions of enzymatic hydrolysis of GL-pretreated substrate and pure cellulose were 2% (w/v) solid content in acetate buffer (0.05 M, pH 4.8), and hydrolyzed for 72 h under 50 °C at 150 rpm/min. In the experiments to explore the pH influence to the enzymatic hydrolysis in the presence of PSS, the buffer with the pH range of 4.0 to 7.0 was prepared by disodium hydrogen phosphate and citric acid. The cellulase dosage (CTec2) was 6.3 FPU/g-glucan for Whatman paper and 13.3 FPU/g-glucan for GL-P. LS and PSS were added to the enzymatic hydrolysis system at the designed amount. After enzymatic hydrolysis for 3, 6, 9, 12, 24, 36, 48, 72 h, enzymatic hydrolysate (1 mL) was extracted and boiled for 10 min, then the supernatant was collected and diluted 5 times to analyze the substrate enzymatic digestibility (SED) or the glucose yield using high-performance liquid chromatography (HPLC, ACQUITY Arc, Waters, USA) equipped with the analytical column BIO-RAD Aminex HPX-87H Column (300 × 7.8 mm) and the guard column CationH Refill Cartridges (30 × 4.6 mm). The test was operated at 55 °C with 5 mM H_2_SO_4_ as mobile phase at the flow rate of 0.6 mL/min.

### Zeta potential of cellulase and PSS

The samples of cellulase, PSS, and their mixtures were incubated at 50 °C and 150 rpm for 2.5 h in the acetate buffer (0.05 M, pH 4.8) to mimic the enzymatic hydrolysis situation. After the temperature dropped to ambient temperature, the samples were taken to measure the zeta potential by Zetasizer Nano-Zs (Malvern, UK).

### Interactions between PSS and cellulase monitored by QCM-D and MP-SPR

The interactions between PSS and cellulase were monitored on an E4 module of quartz crystal microbalance with dissipation monitoring (QCM-D, Biolin Scientific Co. Ltd, Gothenburg, Sweden) and a multi-parametric surface plasmon resonance (MP-SPR, BioNavis Co. Ltd., Tampere, Finland). The procedures for QCM and SPR sensors’ cleaning, cellulase immobilization were described in detail elsewhere [33, 62, 63]. After cellulase immobilization and once the frequency and dissipation curves of QCM-D/SPR stabilized, the PSS solutions (with the concentration of 4.5 mg/mL) were loaded into the chambers until they reached another equilibrium state. At last, acetate buffer was loaded again to rinse the system, and the free PSS loosely bound on the surface of the substrate was removed. The flow rate was controlled at 0.1 mL/min. Each experiment ran at least in duplicate.

Based on the QCM-D overtone data, the thickness, viscositic, and shear moduli of cellulase and PSS films were obtained by Dfind (a software provided with QCM-D instrument by Biolin Scientific Corp.). For MP-SPR data, the recorded full spectra were simulated by the software WinSpall (version 3.02).

## Data Availability

The datasets generated during and analyzed during the current study are available from the corresponding author on reasonable request.

## Abbreviations

LS: Lignosulfonate
PSS: Sodium polystyrene sulfonate
PEG: Polyethylene glycol
CTAB: CetyltrZimethylammonium bromide
BSA: Bovine serum albumin
SPORL: Sulfite pretreatment to overcome recalcitrant of lignocellulose
QCM-D: Quartz Crystal Microbalance with Dissipation monitoring
SPR: Surface plasmon resonance
MP-SPR: Multi-parametric surface plasmon resonance
GL-P: Green liquor-pretreated poplar
KL: Klason lignin
ASL: Acid-soluble lignin
TTA: Total titratable alkali
SED: Substrate enzymatic digestibility
CBM: Carbohydrate-binding module
CD: Catalytic domain
